# Evolution of the availability of Information and Communication Technologies in primary health care in Brazil, 2012 to 2018

**DOI:** 10.1590/1980-549720240021

**Published:** 2024-04-19

**Authors:** Janaína Duarte Bender, Luiz Augusto Facchini, Luís Miguel Velez Lapão, Elaine Tomasi, Elaine Thumé

**Affiliations:** I Universidade Federal de Pelotas – Pelotas (RS), Brazil.; II Universidade Nova de Lisboa – Lisbon, Portugal.

**Keywords:** Primary health care, Health centers, Technology, Information technology, Digital health, Atenção primária à saúde, Centros de saúde, Tecnologia, Tecnologia da informação, Saúde digital

## Abstract

**Objective:**

To verify the evolution of the availability of information and communication technology equipment and inputs in primary health care services that participated in the external evaluation of the Access and Quality Improvement Program in Primary Care and its distribution according to context characteristics social and geographic.

**Methods:**

Cross-sectional study, analyzed the distribution of information and communication technology equipment in basic health units in Brazil, during the three cycles (2012 to 2018) of the Program for Improving Access and Quality in Primary Care. The variables were examined at the municipal level and stratified by geopolitical region. Univariate analysis was performed, using the chi-square test and testing the distributions of exposures among themselves and between the outcome and exposures.

**Results:**

The availability of information and communication technology equipment increased from 9.4% (2012) to 17.5% (2018), with emphasis on the Southeast and South regions, in municipalities with a population size of up to 10,000 inhabitants², with greater family health coverage and high/very high HDI-M. Over the period from 2012 to 2018, basic units joined the program and increased availability of information and communication technologies, such as Internet access, which ranged from 45.2% (n=6,249) to 74.0% (n=21,423), with emphasis on the Northeast region, which increased from 19.1% (n=970) to 58.8% (n=7,087).

**Conclusion:**

Investment in technologies and constant evaluation of primary care in the country is necessary, contributing to its strengthening.

## RESUMO

**Objetivo:**

Verificar a evolução da disponibilidade de equipamentos e insumos de tecnologia de informação e comunicação nos serviços de Atenção Primária à Saúde que participaram da avaliação externa do Programa de Melhoria do Acesso e da Qualidade na Atenção Básica e sua distribuição de acordo com características do contexto social e geográfico.

**Métodos:**

Estudo transversal, que analisou a distribuição de equipamentos de tecnologia de informação e comunicação nas unidades básicas de saúde do Brasil, durante os três ciclos (2012 a 2018) do Programa de Melhoria do Acesso e da Qualidade na Atenção Básica. As variáveis foram examinadas no nível dos municípios e estratificadas por região geopolítica. Realizou-se análise univariada, com o uso do teste χ^2^ e teste das distribuições das exposições entre si e entre o desfecho e as exposições.

**Resultados:**

A disponibilidade do conjunto de equipamentos de tecnologias de informação e comunicação evoluiu de 9,4% (2012) para 17,5% (2018), com destaque às Regiões Sudeste e Sul, em municípios com porte populacional de até 10 mil hab.², com maior cobertura de saúde da família e índice de desenvolvimento humano municipal — IDH-M alto/muito alto. Ao longo do período de 2012 a 2018, houve adesão de unidades básicas ao programa e aumento na disponibilidade de tecnologias de informação e comunicação, como o acesso à internet, que foi de 45,2% (n=6.249) a 74,0% (n=21.423), com destaque para a Região Nordeste com aumento de 19,1% (n=970) a 58,8% (n=7.087).

**Conclusão:**

São necessários o investimento em tecnologias e a constante avaliação da atenção primária no país, contribuindo para seu fortalecimento.

## INTRODUCTION

The availability of health information and communication technologies (HICT) influences the generation, processing, storage, and use of information to improve work processes, management, and technological support, thereby impacting assistance to the population^1^.

Since the 1970s, telecommunications development and innovation have continually reinforced the use of HICTs in the country. During the 1990s, the implementation of information systems focused on assistance, with emphasis on hospital information systems (*sistemas de informações hospitalares* – SIH), live birth information system (*sistema de informações de nascidos vivos* – SINASC), notifiable diseases information system (*sistema de informação de agravos de notificação* – SINAN), and primary care information system (*sistema de informação da atenção básica* – SIAB)^2-5^.

In the 2000s, HICTs were applied to the national health regulatory system (*sistema nacional de regulação em saúde* – SISREG). In 2004, the information and IT policy of the Brazilian Unified Health System (*Sistema Único de Saúde* – SUS) aimed to ensure and regulate the compatible and updated functionality of the information systems (*sistemas de informações* – SIS) SINASC, SIH, SINAN, SIAB of SUS, for example, integrating and articulating with other health systems^6,7^.

With the advancement of HICT in the country, Telehealth became a program of the Ministry of Health (MoH) in 2007, with a focus on healthcare^7^. In 2009, to foster integration with other levels of healthcare, Telehealth expanded its scope to include the training of health professionals in primary care^5,8^.

Primary Health Care (PHC), as care coordinator, is an organized and regionalized strategy designed to address the most pressing health needs of the population, taking into account Brazil’s regional, cultural, socioeconomic, and demographic diversity. Allied with HICT, which comprises a range of technologies that allow access to information through telecommunications — such as computers, hardware, smartphones, tablets, software, internet, telephony, information management systems and databases, storage, and transmission of data^4^ — it is essential to achieving this purpose by facilitating communication between professionals and different points of the Health Care Network (*Rede de Atenção à Saúde* – RAS)^9^.

The availability and use of HICT were investigated by the external evaluation (EA) of the National Program for Improving Access and Quality of Primary Care (*Programa Nacional de Melhoria do Acesso e da Qualidade da Atenção Básica* – PMAQ-AB) in Basic Health Units (*Unidades Básicas de Saúde* – UBS) across Brazil.

Between 2012 and 2018, across the three cycles of the program, there were continuous improvements in infrastructure and availability of resources in Brazilian UBS. The increase in services with internet access and use of electronic medical records played a pivotal role in enhancing professional practice and care for users, albeit remaining challenging for Brazilian PHC^10,11^. Some authors^11,12^ also acknowledge the influence of HICT equipment, such as computers, internet access, and telephones, to facilitate the use of electronic medical records and telehealth on the quality of care, assistance and integration with external systems, impacting communication and coordination within the work process, management, and care in clinical practice^11,12^.

This research aimed to examine the progression of HICT equipment and resources availability in PHC services that participated in the PMAQ-AB external evaluation and to verify their distribution according to characteristics of the social and geographic context.

## METHODS

This is a cross-sectional study, with a quantitative approach. The selection criterion for study participants was the voluntary adherence of health teams to the PMAQ-AB, originated in 2011. External evaluations were carried out in three cycles spanning the years 2012, 2013/2014, and 2017/2018, respectively. The program was an initiative of the Federal Government and consisted of four complementary and continuous phases: adherence and contractualization; development; external evaluation, and re-contracting. PMAQ-AB sought to induce and expand access to improve the quality of basic health care through performance-based financial transfers^13^.

Following the adherence phase of PMAQ-AB, data collection became the responsibility of Federal Higher Education Institutions (*Instituições Federais de Ensino Superior* – IFES), with Universidade Federal de Pelotas among them, under the leadership of the Department of Primary Care of the Ministry of Health. The distribution of municipalities within the territory was shared between IFES, which were responsible for the logistics of moving the interviewers. After mapping intercity routes and devising operational plans, teams of supervisors and interviewers proceeded to collect data at the UBS.

The instruments for data collection were organized into modules and made available electronically on tablets to health professionals (nurses, doctors, or dentists) at the UBS. Immediately after collection, the information was automatically transferred to the database on a national server. Each institution overseeing the collection conducted an initial consistency analysis of the database, thereby serving as the primary data sources for the study.

In this study, module I — “Observation in the Basic Health Unit” — was used, focusing on the section “Information Technology and Telehealth Equipment in the Health Unit”.

Thus, the variables used in cycles I, II, and III of PMAQ-AB were those available at the UBS:

- Computer (Yes/No)
- Camera (Yes/No)
- Television (Yes/No)
- Internet access (Yes/No)

The variables telephone, smartphone, tablet, microphone, printer, speaker, and data projector were not considered due to their low presence in services. With the selected variables, a score was formulated, representing the sum of positive responses to equipment and input variables in each UBS. Subsequently, this score was dichotomized and those UBS with all items were considered to have an adequate HICT structure.

The independent variables were examined at the municipal level and stratified by geopolitical region:

- Estimated number of inhabitants for 2012, 2014, and 2018 (up to 10,000; 10,001–30,000; 30,001–100,000; 100,001–300,000; more than 300,000);
- HDI-M according to the United Nations Development Program (<0.555 = low; 0.555–0.699 = medium; 0.700–0.799 = high; 0.800–1.000 = very high);
- Population coverage of the Family Health Strategy (FHS) in 2012, 2014, and 2018 (up to 50%; 50.1 to 75.0%; 75.1 to 99.9%; 100%);
- Geopolitical region (North; Northeast; Central West; Southeast; South).

Univariate analysis was performed using the χ^2^ test and the distributions of exposures were tested both among themselves and between the outcome and exposures. To analyze the evolution of the outcome during the EA period, the p-value was calculated. The data were analyzed using the STATA statistical software, version 15.1.

The studies were submitted and approved by the Research Ethics Committees (*Comitês de Ética em Pesquisa* – CEP) involved. In cycle I, approval was granted by the CEP of Universidade Federal de Pelotas under opinion No. 38/2012, on May 10, 2012. In cycle II, approval was obtained from the CEP of Universidade Federal de Goiás under opinion No. 487055, on December 2, 2013. Cycle III was approved by the CEP of Universidade Federal de Pelotas under opinion No. 2.453.320, on December 27, 2017.

## RESULTS

PMAQ-AB external evaluation encompassed a total of 13,842 UBS in cycle I, 24,055 in cycle II, and 28,939 in cycle III across the country. The availability of the set of HICT equipment in UBS throughout the three cycles was 9.4, 9.6, and 17.5%, respectively.

The South Region stood out with the highest proportions of HICT equipment availability, showing continuous growth, going from 20.2% in cycle I to 24.5% in cycle II, and 35.8% in cycle III. The North Region remained the one with the lowest availability of HICT equipment, with 3.7% in cycle I, 2.1% in cycle II, and 6.0% in cycle III (Table 1).

**Table 1:** Distribution of Basic Health Units and availability of all information and communication technology equipment* according to the characteristics of the municipalities. National Program for Improving Access and Quality of Primary Care, 2012, 2013/14, and 2017/18.

	Cycle I 2012 n (%)		Cycle II 2013/14 n (%)		Cycle III 2017/18 n (%)	
	n (%) UBS^†^	n (%) HICT*	n (%) UBS^†^	% HICT*	n (%) UBS^†^	% HICT*
Total Brazil	13,842 (100)	1,294 (9.4)	24,055 (100)	2,306 (9.6)	28,939 (100)	5,064 (17.5)
**p-value**	0.000	0.000	0.000	0.000	0.000	0.000
Region						
North	803 (5.8)	30 (3.7)	1,429 (6.0)	30 (2.1)	1,920 (6.6)	117 (6.0)
Northeast	5,087 (36.8)	95 (1.9)	9,704 (40.3)	512 (5.3)	12,048 (41.6)	1,426 (11.8)
Southeast	4,623 (33.4)	607 (13.1)	7,165 (29.8)	691 (9.6)	8,331 (28.8)	1,481 (17.8)
South	2,391 (17.3)	482 (20.2)	3,607 (15.0)	884 (24.5)	4,160 (14.4)	1,490 (35.8)
Central-West	938 (6.8)	80 (8.5)	2,150 (8.9)	189 (8.8)	2,480 (8.6)	550 (22.2)
**p-value**	0.000	0.000	0.000	0.000	0.000	0.000
Size (inhab.)^‡^						
Up to 10,000	2,362 (17.1)	578 (24.5)	3,563 (14.8)	765 (21.5)	4,264 (14.9)	1,257 (29.5)
10,001 to 30,000	4,508 (32.6)	248 (5.5)	7,844 (32.6)	656 (8.4)	9,624 (33.8)	1,570 (16.3)
30,001 to 100,000	3,326 (24.0)	90 (2.7)	6,247 (26.0)	343 (5.5)	7,570 (26.6)	1,107 (14.6)
100,001 to 300,000	1,645 (11.9)	83 (5.1)	3,085 (12.8)	195 (6.3)	3,398 (11.9)	460 (13,5)
More than 300,000	2,001 (14.5)	295 (14.7)	3,312 (13.8)	347 (10.5)	3,653 (12.8)	624 (17.0)
**p-value**	0.000	0.000	0.000	0.000	0.000	0.000
HDI-M^§^						
Low/medium	10,968 (79.2)	789 (7.2)	13,329 (56.6)	973 (7.3)	12,799 (45.1)	1,608 (12.6)
High/very high	2,874 (20.8)	505 (17.6)	10,212 (43.4)	1,273 (12.5)	15,605 (54.9)	3,373 (21.6)
**p-value**	0.000	0.000	0.000	0.000	0.000	0.000
FH coverage^//^						
Up to 50%	3,165 (22.9)	284 (8.9)	3,754 (15.6)	301 (8.0)	7,601 (26.3)	1,150 (15.1)
50.1 to 75%	3,344 (24.2)	295 (8.8)	4,197 (17.5)	273 (6.5)	6,560 (22.7)	1,030 (15.7)
75.1 to 99.9%	3,064 (22.1)	235 (7.7)	4,899 (20.4)	503 (10.3)	6,196 (21.4)	1,104 (17.8)
100%	4,269 (30.8)	480 (11.2)	11,163 (46.5)	1,226(11.0)	8,574 (29.6)	1,779 (20.7)

*Number of UBS that had all the equipment (computer, camera, television, and internet access);

^†^Total number of basic health units participating in the National Program for Improving Access and Quality of Primary Care;

^‡^Population size;

^§^Municipal Human Development Index;

^//^Family Health Coverage.

With the exception of services located in municipalities with more than 300 thousand inhabitants, all others showed an increase in the availability of HICT equipment over the years. In cycles I and II, services in municipalities with 30,001 to 100,000 inhabitants showed lower availability of the set of HICT equipment, with 2.7 and 5.5%, respectively. In cycle III, UBS located in municipalities with 100,001 to 300,000 inhabitants presented the lowest availability of equipment (13.5%) (Table 1).

Health units located in municipalities with high and very high HDI-M consistently presented approximately twice as much availability of the set compared to units in municipalities with low and medium HDI-M across all three cycles. In the latter municipalities, the increase in the availability of ICT equipment was modest, reaching only 12.6% of UBS in the third cycle (Table 1).

Throughout the three cycles, there was a higher prevalence of HICT equipment availability among UBS located in municipalities with 100% FHS coverage, increasing from 11% in cycles I and II to 20.7% in cycle III (Table 1).

Among the equipment investigated, the computer was the most prevalent item in UBS, with 64.0% in cycle I, 69.6% in cycle II, and 89.3% in cycle III. Television came next, showing a slight decline over the period: 61.7% in cycle I, 56.7% in cycle II, and 58.8% in cycle III. The camera was the equipment with the lowest availability, ranging from 13.0% in cycle I to 14.9% in cycle II and 25.7% in cycle III. Internet access was increasingly available throughout the three cycles, with 45.2% in cycle I, 50.1% in cycle II, and 74.0% in cycle III (Table 2).

**Table 2:** Availability of information and communication technology equipment according to the characteristics of the municipalities*. National Program for Improving Access and Quality of Primary Care, 2012, 2013/14 and 2017/18.

	Computer			Camera			Television			Internet access		
	Cycle I 2012 n (%)	Cycle II 2014 n (%)	Cycle III 2018 n (%)	Cycle I 2012 n (%)	Cycle II 2014 n (%)	Cycle III 2018 n (%)	Cycle I 2012 n (%)	Cycle II 2014 n (%)	Cycle III 2018 n (%)	Cycle I 2012 n (%)	Cycle II 2014 n (%)	Cycle III 2018 n (%)
Total Brazil	8,855 (64.0)	16,741 (69.6)	25,838 (89.3)	1,750 (13.0)	3,573 (14.9)	7,431 (25.7)	8,542 (61.7)	13,631 (56.7)	17,012 (58.8)	6,249 (45.2)	12,055 (50.1)	21,423 (74.0)
**p-value**	0.000	0.000	0.000	0.000	0.000	0.000	0.000	0.000	0.000	0.000	0.000	0.000
Region												
North	419 (52.2)	816(57.1)	1,742 (90.7)	71 (8.8)	89 (6.2)	213 (11.1)	416 (51.8)	619 (43.3)	949 (49.4)	190 (23.7)	365 (25.5)	1,054 (54.9)
Northeast	1,841 (36.2)	4,950 (51.0)	9,626 (79.9)	234 (4.6)	1,200 (12.4)	2,708 (22.5)	2,344 (46.1)	4,118 (42.4)	5,804 (48.2)	970 (19.1)	2,743 (28.3)	7,087 (58.8)
Southeast	3,661 (79.2)	5,908 (82.5)	7,938 (95.3)	727 (15.7)	874 (12.2)	1,939 (23.3)	3,525 (76.3)	5,143 (71.8)	5,791 (69.5)	2,683 (58.0)	4,591 (64.1)	7,113 (85.4)
South	2,193 (91.7)	3,348 (92.8)	4,113 (98.9)	587 (24.6)	1,095 (30.4)	1,849 (44.5)	1,681 (70.3)	2,526 (70.0)	2,958 (71.1)	1,899 (79.4)	3,012 (83.5)	3,994 (96.0)
Central-West	741 (79.0)	1,719 (79.9)	2,419 (97.5)	131 (13.9)	315 (14.7)	722 (29.1)	576 (61.4)	1,225 (56.9)	1,510 (60.9)	507 (54.1)	1,344 (62.5)	2,175 (87.7)
**p-value**	0.000	0.000	0.000	0.000	0.000	0.000	0.000	0.000	0.000	0.000	0.000	0.000
Size (inhab.)												
Up to 10,000	1,720 (72.8)	2,788 (78.3)	3,916 (91.8)	670 (28.4)	974 (27.3)	1,522 (35.7)	1,709 (72.4)	2,440 (68.5)	3,156 (74.0)	1,486 (62.9)	2,385 (66.9)	3,606 (84.6)
10,001 to 30,000	2,473 (54.9)	5,084 (64.8)	8,325 (86.5)	411 (9.1)	1,181 (15.1)	2,431 (25.3)	2,455 (54.5)	3,976 (50.7)	5,437 (56.5)	1,547 (34.3)	3,280 (41.8)	6,672 (69.3)
30,001 to 100,000	1,874 (56.3)	4,019 (64.3)	6,654 (87.9)	182 (5.5)	646 (10.3)	1,737 (22.9)	1,826 (54.9)	3,112 (49.8)	4,071 (53.8)	1,180 (35.5)	2,644 (42.3)	5,182 (68.5)
100,001 to 300,000	1,123 (68.3)	2,212 (71.7)	3,132 (92.2)	137 (8.3)	320 (10.4)	789 (23.2)	1,024 (62.3)	1,834 (59.5)	1,919 (56.5)	732 (44.5)	1,585 (51.4)	2,561 (75.4)
More than 300,000	1,665 (83.2)	2,635 (79.6)	3,400 (93.1)	350 (17.5)	452(13.7)	891 (24.4)	1,528 (76.4)	2,268 (68.5)	2,189 (59.9)	1,304 (65.2)	2,159 (65.2)	3,087 (84.5)
**p-value**	0.000	0.000	0.000	0.000	0.000	0.000	0.000	0.000	0.000	0.000	0.000	0.000
HDI-M												
Low/medium	6,208 (56.6)	7,649 (57.4)	10,344 (80.8)	1,156 (10.5)	1,811 (13.6)	2,805 (21.9)	6,280 (57.3)	6,423 (48.2)	6,641 (51.9)	4,051 (36.9)	4,612 (34.6)	7,544 (58.9)
High/very high	2,647 (92.1)	8,675 (84.9)	14,978 (95.9)	594 (20.7)	1,688 (16.5)	4,517 (28.9)	2,262 (78.7)	6,845 (67.0)	10,049 (64.4)	2,198 (76.5)	7,127 (69.8)	13,475 (86.4)
**p-value**	0.000	0.000	0.000	0.000	0.000	0.000	0.000	0.000	0.000	0.000	0.000	0.000
FH coverage												
Up to 50%	2,458 (77.7)	3,045 (81.1)	7,220 (94.7)	382 (12.1)	454 (12.1)	1,702 (22.4)	2,161 (68.3)	2,400 (63.9)	4,532 (59.6)	1,800 (56.9)	2,440 (65.0)	6,247 (82.2)
50.1 to 75%	2,234 (66.8)	2,937 (69.9)	5,807 (88.5)	399 (11.9)	426 (10.2)	1,565 (23.9)	2,054 (61.4)	2,408 (57.4)	3,714 (56.6)	1,591 (47.6)	2,183 (52.0)	4,782 (72.9)
75.1 to 99.9%	1,870 (61.0)	3,494 (71.3)	5,546 (89.5)	321 (10.5)	749 (15.3)	1,633 (26.4)	1,797 (58.7)	2,739 (55.9)	3,537 (57.1)	1,312 (42.8)	2,507 (51.2)	4,584 (73.9)
100%	2,293 (53.7)	7,237 (64.8)	7,277 (84.9)	648 (15.2)	1,940 (17.4)	2,528 (29.5)	2,530 (59.3)	6,060 (54.3)	5,224 (60.9)	1,546 (36,2)	4,903 (43.9)	5,803 (67.7)

HDI-M: municipal human development index; FH: Family health.

* Total number of Basic Health Units that reported the availability of at least one piece of equipment in operation.

The following analysis examines the availability of each equipment in UBS by region, population size, HDI-M, and family health coverage across the three PMAQ-AB cycles.

Computer availability increased in all regions, with the most significant growth observed in the North and Northeast Regions, nearly doubling the equipment registered in 2012 by 2018. In the North, availability rose from 52.2 to 90.7% and in the Northeast, from 36.2% to 79.9% over the three cycles. There was also an upward evolution in computer availability in all population size categories. UBS located in municipalities with 10,001 to 100,000 inhabitants were responsible for the largest increases in this availability, as this was a reality for less than half of the services in the first cycle and reached more than 85% in the third cycle. Similar trends were observed in UBS located in municipalities with low and medium HDI-M, where availability increased 56.6% in cycle I, 57.4% in cycle II, and 80.8% in cycle III. The growth in computer availability at UBS was also observed in all FHS coverage categories, with emphasis on services located in municipalities with 100% coverage, which went from 53.7% in cycle I to 84.9% in cycle III (Table 2).

Cameras were more prevalent in the South Region throughout the three cycles, showing growth from 24.6% in 2012 to 44.5% in 2018. In the Central-West Region, evolution occurred in the second and third cycles, from 14.7 to 29.1%, respectively. Regarding population size, the availability of cameras was highest in municipalities with up to 10 thousand inhabitants, reaching 28.4% in cycle I, 27.3% in cycle II, and 35.7% in cycle III. Additionally, the availability of cameras in municipalities with high and very high HDI-M maintained prominent in all three cycles, with 20.7, 16.5, and 28.9%, although there was a drop in 2014. The greater availability of cameras was observed in UBS with 100% family health coverage, going from 15.2% in cycle I to 29.5% in cycle III. The UBS with less availability of cameras varied during the evolution of the three cycles (Table 2).

The South Region had greater television availability across the three cycles, remaining between 70.0 and 71.1%. The Northeast Region had the lowest television availability, ranging from 42.4% in cycle II to 48.2% in cycle III. Television availability varied across cycles: in cycle I, the highest prevalence was in municipalities with more than 300 thousand inhabitants, at 76.4%; in cycle II, in municipalities with up to 10 thousand inhabitants and those with more than 300 thousand inhabitants, which presented 68.5%; in cycle III, municipalities with up to 10 thousand inhabitants represented, with 74%, the highest availability of televisions. Although high and very high HDI-M areas predominated in television availability, there was a declining trend across the three cycles, with figures of 78.7%, 67.0%, and 64.4%, respectively. In UBS with up to 50% FHS coverage, the availability of televisions declined from 68.3% in cycle I to 59.6% in cycle III. In cycle II, UBS with 100% FHS coverage had only 54.3% television availability (Table 2).

Internet access stood out in the South Region, increasing from 79.4% in 2012 to 96.0% in 2018. The North and Northeast Regions had the lowest rates among the three cycles. In cycle 1, municipalities with more than 300 thousand inhabitants had greater availability of internet access, at 65.2%, but in cycles II and III, municipalities with up to 10 thousand inhabitants had higher availability, with 66.9 and 84.6 %, respectively. Availability of internet access in municipalities with high and very high HDI-M prevailed, with 76.5% in cycle I, 69.8% in cycle II, and 86.4% in cycle III. The highest availability of internet access was in UBS with up to 50% of FHS coverage, with an upward evolution from 56.9% in cycle I to 82.2% in cycle III. However, UBS with 100% coverage had lower internet access availability across all three cycles, with 36.2, 43.9 and 67.7%, respectively (Table 2).

## DISCUSSION

In Brazil, most healthcare facilities use computers and have internet access. However, regions experiencing connectivity issues dace challenges in accessing HICT. A study^14^ indicated that 51.2% of UBS had at least one computer, and 35.4% had internet access^14^. Studies report that, in cycle I of PMAQ-AB, only 13.5% of teams had optimal infrastructure, systems, and information use conditions. Despite the inclusion of new UBS in the second cycle of PMAQ-AB, only 8.5% were considered computerized, meaning they had a set of equipment consisted of a computer, camera, telephone, and internet access — even though the proportion of UBS with internet access increased from 45.1% in cycle I to 74.0% in cycle III^10,15-17^.

Studies suggest that HICT have great potential for health care. However, due to their limited availability in UBS, they become a gap that requires further investment. Factors such as regional inequalities and shortage of human resources also contribute to precarious conditions in the structure and quality of HICTs in UBS^14,17,18^.

The availability of HICTs is concentrated in the South and Southeast Regions, in municipalities with smaller population sizes, high/very high HDI-M, and where FH coverage is 100%. These associations underscore the importance of universal HICT availability in UBS for the effective functioning of SUS information systems, with notable inequality between regions and greater fragility in the North and Northeast^19^.

One way to elucidate this discrepancy is through Brazil’s extensive economic, social, cultural, and territorial disparities. The Brazilian territorial distribution, represented here by population size, exerts influence on the conditions of healthcare management conditions, socioeconomic status, and health situation of the population, spatial configuration, and structuring of health services^20,21^.

The total of 5,566 Brazilian municipalities are classified as adjacent and remote rural areas with difficult access, mainly in the North and Northeast regions, rendering them more susceptible to technology availability challenges. Internet access is often non-existent or unstable in these areas^22^. A study addresses this deficit of HICT in these workplaces due to a gap in primary and outpatient care. A though hospitalizations are not common in these locations, they still require technological equipment such as computers, cameras, televisions, and internet access^23^.

Despite the increasing reliance on the internet and the pursuit for technological advancements, access remains one of the main barriers to HICT. This fact is in line with a Brazilian study that demonstrated the evaluation of actions in the three cycles of PMAQ-AB and presented to professionals and managers the potentialities and weaknesses concerning access and service quality, reinforcing the importance of making adaptations and policy formulations, professional training, continuing education, and research efforts^10,19^.

The incorporation of HICTs into the country’s primary care system is in constant development, as evidenced by the evolution of HICTs with PMAQ-AB and the Support Program for Computerization and Qualification of PHC data, established by Ordinance No. 2.983/2019^10,24,25^. However, this process is still in its early stages, with more accelerated advancements observed in large urban centers or municipalities with higher socioeconomic levels. In line with our study, the author highlights that the Southeast Region boasts a higher quantity of HICT equipment, while the North Region faces a scarcity of such equipment^24^.

The shortage of HICT resources has been observed since before 2020, a situation that has not yet been resolved during the COVID-19 pandemic. This period presented heightened the demand for HICTs, as they became the primary tools for operating services, managing, processes, access, and healthcare, influencing new habits in the sector. In this scenario, the use of the internet, computers, telephones, and cameras to access electronic medical records, teleservices, teleconsultations and the use of applications highlighted the importance of HICT^10,26-28^.

Other studies indicate that addressing the shortage of HICTs, particularly internet access, electronic medical records, and the availability of computers and cell phones, depends on a plan that extends to approximately 30 thousand UBS to universalize access to all resources in SUS^5,10^.

Just as one study^24^ reports an incipient process of HICT implementation, another research^29^ addresses the implementation of HICT as precarious, particularly concerning the informational continuity between primary care and specialized care. The authors also emphasize the coordination between services and actions to improve the provision of comprehensive and synchronized care, through enhancing PHC with network integration strategies and investment in HICT. In line with this perspective, authors^30^ showed that HICTs provide the articulation of health actions, strengthening the care model in PHC with the expansion of FHS coverage. This prompts reflection on the higher prevalence of HICTs observed in UBS with such coverage, as demonstrated in the present study^24,29,30^.

Therefore, there is a need for significant investments to achieve full availability of HICTs, aiming to expand access and improve the comprehensiveness and equity of PHC in Brazil^26-28^.

We acknowledge that HICT encompasses a wide range of technological equipment, as well as the need for human resources. This aspect can be regarded as a limitation of this study, alongside the scarcity of literature on the availability and usage of HICT equipment in PHC. Additionally, the exclusion of equipment that was notably missing in the three cycles of external evaluation, such as stabilizers, smartphones, data projectors, telephones, tablets, and teleconferencing equipment, prevented us from analyzing their evolution.

This study illustrated the progression of HICT equipment and input availability in PHC services that partook in the PMAQ-AB external evaluation and its association with municipal characteristics. HICTs are a growing means of learning due to the expansion of internet access and the possibility of overcoming geographical barriers, thereby providing democratization of access. The findings of this study highlight the evolution of the supply of ICTs, which is reflected in the potential qualification of PHC, as they are an important tool for health services and policies, strengthening the work process of professionals, qualifying health services and improving attention to system users.

The participation of UBS throughout the three cycles reinforced the need for investments in HICT, which remains a significant challenge. Greater efforts are required to address inequalities in the availability of HICTs, as they contribute to strengthening PHC. Sustaining investments and evaluating PHC as a key provider of care within the SUS service network are also recommended.
